# Fee exemption for caesarean section in Morocco

**DOI:** 10.1186/0778-7367-70-3

**Published:** 2012-01-03

**Authors:** Issam Bennis, Vincent De Brouwere

**Affiliations:** 1 Institut National d'Administration Sanitaire, Ministère de la Santé, Rabat, Morocco; 2 IRD, UMR912, Marseille, France; Institut National d'Administration Sanitaire, Rabat, Morocco; and Institute of Tropical Medicine, Antwerp, Belgium

**Keywords:** Access to care, caesarean section, cost of care, Morocco, maternal mortality

## Abstract

Financial barriers are an important obstacle for access to emergency obstetric care and a contributing factor to too slow a reduction in the level of maternal mortality. In Morocco, in 2009, a fee exemption policy for delivery and caesarean section was implemented in public maternity hospitals. As in most countries where a fee exemption policy has been implemented, fee exemption is considered synonym to free care. However, other direct costs may subsist. The objective of this study was to get an estimate of the actual cost of caesarean sections from the patients' perspective.

This study was carried out in April 2010 in the three public hospitals in Fez. We carried out semi-structured interviews among a sample of 100 women who gave birth by caesarian section in the public hospitals in Fez. The results showed that households paid between US$169 (95% Confidence Interval (CI): 153, 185) at the provincial and regional hospitals, and US$291 (95% CI: 224-359) at the university hospital (UH) where the fee exemption was not applied. The direct cost of a caesarean was mainly influenced by the price of the drugs the families bought, the invoice paid at UH, and the transport. Finally, although the fee exemption policy for caesareans has probably reduced the total cost for households who did not have access to a poverty card, it has not led to 'truly' free caesarean deliveries.

## Introduction

The reduction of maternal mortality is one of the Millennium Development Goals (MDGs). It received a lot of media attention, largely because the international community realizes that despite the efforts of the past 20 years, this MDG will not be achieved by 2015 [1].

Although the causes of maternal deaths and the medical means to avoid them are well-known, the challenge in most developing countries is to find the right strategic balance between incentives of supply and demand [2]. Lack of access to quality care, particularly the lack of financial access, is a constant headache in poor countries where households are sometimes forced to borrow heavily to pay for transportation and obstetric care if they manage to reach the hospital on time [3,4].

In Morocco, a study based on the 1995 Demographic and Health Survey and the Service Availability Module data showed the high negative impact of user fees on utilization of maternity care by the poorer households [5]. Later, in 2004, the Population and Health Family Survey (PHFS) reported that lack of money was a major obstacle to access maternity care for 74% of women interviewed [6].

The reduction of maternal mortality became a national priority in Morocco in 2008, following the results of the 2003-04 PHFS study which showed a stagnation in maternal mortality ratios [6]. A number of measures were introduced to try and reduce maternal deaths to 50 per 100,000 live births by 2012 [7]. One of them was to make deliveries free of charge in public hospitals. This meant that the following services are free for every woman: delivery (vaginal delivery or a caesarean section, essential lifesaving medicines, laboratory examinations, ultrasonography, radio, and blood transfusion), obstetric complication around childbirth (pre-eclampsia, eclampsia, sepsis, etc.), hospital stay (including intensive care unit if required), transport from a peripheral maternity to a hospital maternity (mother and/or newborn), and in rural areas transport from home to the hospital in case of emergency. The ministerial decision whereby the delivery fee in public hospital was abolished was introduced early 2009. However, the fee exemption policy cannot be considered as truly free as long as some hidden costs persist. These hidden costs may be high enough to make patients delay their decision to go to hospital, sometimes with dramatic results, or may lead to catastrophic expenditures for households [8].

The objective of this study is to get an estimate of the actual cost paid in the Fez public hospitals by patients who needed a caesarean section.

## Method

### Context

The principal investigator (PI) selected the three public referral hospitals in Fez district, Morocco, where he worked. These hospitals serve the city of Fez and the neighboring provinces (Taounat, Moulay Yacoub, Sefrou, Boulmane), a population of 1.7 million inhabitants (5.4% of the total Moroccan population) [9].

Two of the three hospitals, the provincial hospital (PH) and the regional hospital (RH), fall under the SEGMA system (governmental service managed autonomously) and the direct authority of the Ministry of Health (MOH). The third hospital, the university hospital (UH), is managed as an autonomous public institution. In 2009, the two SEGMA hospitals carried out 7952 vaginal deliveries and 1247 caesarean sections, while the UH carried out 4137 vaginal deliveries and 1002 caesareans.

Besides these three, there are a number of private hospitals but the average cost of a caesarean in the private sector is higher than US$1000, which makes it inaccessible for the poor; moreover the decision to provide fee exemption for caesarean deliveries does not apply to the private sector.

Normal (vaginal) deliveries have been free in the public small maternities since decades and are free since 2009 in public hospitals. Before the fee exemption policy, normal delivery had to be paid at a flat fee of $64 in public hospitals, except if the woman could bring the evidence of her indigence (thanks to a poverty card obtained from the local authority, a sometimes long and difficult process) or if she was covered by a health insurance (a small minority of the population). For a caesarean section, women had to pay a lump sum of $283 [10]. The additional costs for a vaginal delivery, due for instance to prescribed drugs, have not been investigated although these costs, in the experience of the investigators, were kept low. Tariffs have been abolished by the fee exemption policy.

Initially, the MOH distributed delivery and caesarean kits in which the essential medicines and consumables were packed to all the public facilities. Later, in 2010, public hospitals got an additional budget to order the required essential drugs and consumables for deliveries and C-sections. The list of drugs is however limited to the WHO essential ones and usually are generic medicines.

### Data collected

The information about household expenditure was collected between 1^st ^and 30^th ^April 2010, as part of the practical training of the PI, from a sample of 100 women who gave birth by caesarean section in one of the three hospitals: 68 in the RH (all women delivered by caesarean section in April 2010), 16 in the PH (interviews taken from all the women delivered by caesarean in the week of 16^th ^to 23^rd ^April 2010) and 16 in the UH (interviews of all the women delivered by caesarean from 19^th ^to 30^th ^April 2010).

The women were interviewed together with their family at the moment they left the hospital (26 patients, 56 husbands and 18 accompanying persons (carers other than the husband)). The information about the direct cost of the caesarean section was collected through a questionnaire with a mix of open and closed questions. The first part, designed for the women and/or their carers immediately after the delivery consisted of face-to-face interviews. It was carried out inside the hospital and aimed at gathering information about the pregnancy and about the degree of knowledge about the fee exemption system for caesareans; and at putting the interviewed person (the husband in most cases) at ease. During this first interview the investigator also asked the consent of the interviewee to continue the interview at a later stage - once the patient had left the hospital - through a telephone survey. This latter part of the interview primarily focused on drug costs after leaving the hospital and the cost of informal payments (bribes). If the families did not have a mobile phone, they were interviewed outside the hospital on the last day of the women's stay (8 cases).

We calculated the price of drugs and pharmaceutical products prescribed for each patient from the time they were admitted to the hospital until leaving, based on prescriptions they showed the researcher. The Moroccan currency (MAD) was converted into US$ at a rate of US$ 0.127 for 1 MAD. The direct interview with the women and/or their carers enabled us to estimate the amount of informal payments and the cost of extra food. The families were also asked to list the total travel cost and the opportunity costs for the wife and her carer. For the woman, only the trip that ended in her admission to the hospital was taken into account. The travel cost of the main carer (return journey between home and the hospital) was multiplied by the number of visits during the hospital stay. Finally, we added the cost of the woman's return journey on exiting the hospital. The extra cost for food and the opportunity costs were calculated only for the women who had a caesarean section but not for their companions. All the data from the questionnaires have been transcribed in variables, encoded in Excel 2007, and transferred to SPSS version 16.0 for statistical analysis. For some variables, we have calculated measures of frequency, central tendency and dispersion.

### Ethical considerations

The interviews were carried out by a male doctor, who introduced himself as a Masters student. The women and their family were informed about the study objectives, that they could refuse to participate, were free not to reply to certain questions or interrupt the interview at any moment without this having an influence on their quality of care. It was explained that their honest reply to the questions would help get a better understanding of the fee exemption actual process. They were assured that their replies would be treated confidentially and that their name would not be mentioned on any of the forms. At the end of this introduction, they were asked again about their consent to participating in the study. If they consented they were asked for a telephone number where they could be reached once they had left the hospital.

In Morocco, the directors of the public hospitals guarantee the protection of hospitalized patients. A written authorization giving permission to carry out the study was obtained for each of the hospitals involved in the study.

## Results

### Direct expenditure of households

Costs include the price of drugs bought for the operation and the cost of prescribed drugs upon leaving, transport, opportunity costs, extra food and the informal payments.

Of the 100 women interviewed, 14 had bought pharmaceutical products^a ^before the caesarean section costing between US$10 to 68. Most of the women (95%) bought pharmaceuticals after the intervention at an average cost of US$85 (95% CI: 76-96) at the SEGMA hospitals and US$74 (95% CI: 51-98) at the university hospital. For the regional hospital, three families out of 68 said they spent no money on drugs since they had received free samples from the hospital staff.

The transport cost was not statistically higher for women who stayed at the university hospital although the average cost was US$68 (95% CI: 42-94) for the UH compared to US$52 (95% CI:45-59) for the SEGMA hospitals (Mann-Whitney U test, p = 0.13). In our total sample, only 12 women (out of 100) had the benefit of an ambulance for all or part of their journey to the hospital. Four women used an ambulance but had to contribute to the cost (they confirmed having paid between US$6 to $32 for a local ambulance since they did not use the free of charge MOH ambulance). The average cost of this first journey was between US$6 (95% CI: 4-8) to reach the SEGMA hospitals and US$16 (95% CI:7-24) to reach the university hospital.

Less than 50% of the people we interviewed said they made informal payments. Fifty-one people said they did not make informal payments and 10 refused to answer this question. Among those who made informal payments, the cost varied between US$4 and US$51. Proportions of families having given informal payments varied from 25% in UH, to 49% in RH but were not statistically different. The majority of those interviewed assured us that they willingly paid this sum. They did so either because they wanted the staff to share in their joy on the occasion of the birth, or to help non-medical staff (security agents and cleaning ladies) since they get a very low salary. Another reason is that by paying in this way the staff become more attentive or allow them to visit their wife in hospital outside official visiting hours, or they allow more visitors by the bedside than is officially permitted.

Food and opportunity costs also represent an extra cost for the family. Seventy-seven people spent money to buy extra food during the women's stay in hospital after the operation, the majority of these expenses were made from the third day after admission. The cost of extra food was US$16 (95% CI: 11-20) for the SEGMA hospitals and US$5 (95% CI: 0-10) for the university hospital. The majority of carers (64%) and 100% of women saw no reduction in their financial resources as a result of their temporary cessation of work due to hospitalization. For the 32 carers involved, the costs amounted to US$24 (Standard deviation US$14) on average.

The sum paid by households to the Billing service of the hospital was zero US$ for the RH and the PH. Women in the UH, whether or not referred, had to pay at least US$127 for their caesarean section although a referred woman from an RH should not pay according to the policy. The poverty card (certificate issued by the local authority that officially recognizes a household as 'poor') was systematically refused by the UH, although it was accepted for a stay in an Intensive Care Unit, the medical department or surgery.

In summary, the direct cost of a caesarean section was on average US$291 (95% CI: 224-359) in the UH and US$169 (95% CI: 153-185) in the SEGMA hospitals (Table 1).

**Table 1:** Direct cost of a caesarean section for families in the Fez hospitals

	SEGMA hospitals			University hospital		
	
	Nr respondents	Mean (95% CI)	Minimum/Maximum	Nr respondents	Mean (95% CI)	Minimum/Maximum
Invoice paid	84	$0	$0/$0	16	$138 (98-178)	$0*/$304

Pharmaceutical products and consumables	82	$85 (76-96)	$0/$228	15	$74 (51-98)	$13/$158

*Transport cost 1^st ^journey*	*84*	*$6 (4-8)*	*$0/$63*	*16*	*$16 (7-24)*	*$0/$44*

*Transport cost (after 1^st ^journey)*	*84*	*$46 (40-52)*	*$0/$121*	*16*	*$52 (28-78)*	*$5/$162*

Total transport cost	84	$52 (45-59)	$0/$163	16	$68 (42-94)	$5/$175

Tips and unofficial payments	74	$11 (7-14)	$0/$51	16	$3 (0-7)	$0/$19

Cost of extra food	84	$16 (11-20)	$0/$127	16	$5 (0-10)	$0/$32

Opportunity cost	83	$8 (5-11)	$0/$63	15	$7 (0-15)	$0/$38

**Global Cost of Caesarean**	**84**	**$169** **(153-185)**	**$26/$376**	**16**	**$291** **(224-359)**	**$138/$616**

* women who paid $0 were covered by a health insurance which was charged $127

### Coping strategies

**Figure 1:**
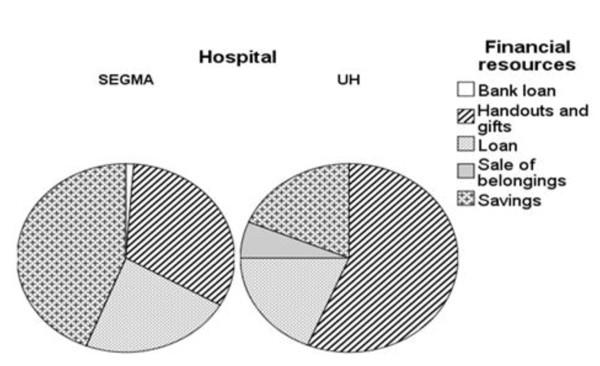
**Family coping strategies to cover expenses linked to a caesarean section in Fez SEGMA hospitals and the University Hospital (UH)**.

It was not possible to exactly quantify the indirect cost of a caesarean delivery due to the unreliability of the data. It was nevertheless possible to determine the number of people who declared having had to ask for handouts and loans to cover the expenses of a caesarean section which in most cases was not foreseen. Families had to fall back on handouts and loans in nearly 75% of 16 cases for the UH against 55% of 84 cases in the SEGMA hospitals. Only one person from the Moulay Yacoub province had to sell his carpet, his chickens and his radio to cover the hospital fee in the UH (Figure 1). Three households were obliged to extend the woman's stay in hospital in order to find the money to pay the invoice when they discovered that the caesarean section was not for free in the UH and that their poverty card was refused.

We have not been able to assess the physical consequences of this operation for women who delivered in this manner, nor the reduction in their ability to work, even at home. However, women having had a previous caesarean section said they did not have the same physical energy to carry out their work as before.

## Discussion

This study was carried out in three hospitals, which according to the experience of the researchers operate in the same manner as other public hospitals in Morocco. However, we do not know whether they are close to the average or whether there is a divergence; it is thus not possible to generalize our results to all the Moroccan public hospitals. A representative sample from the regions would have allowed us to confirm the effect of the fee exemption policy. Also, the interviews were carried out over a very short period and do not allow us to confirm that the levels and ratios of the different expenses are similar over the year.

The Moroccan fee exemption policy has certainly shown some efficacy since it made the medical and surgical acts free for every woman in the public hospitals. We do not know if it is an advantage for the poor who benefited in the past from the poverty card to cover all the expenses charged by the hospital. As it is not easy to get a poverty card, the universal fee exemption certainly has had the effect of simplifying the procedure and allowing the less poor to save 283$. It also showed its limitations since it does not seem to apply to the Fez UH, although the MOH and the UHs agreed to apply a fee exemption when women are referred from a regional hospital. This may need further investigation.

The extra costs for the purchase of medicines which are not included in the caesarean « kit « are not statistically different at the UH compared to the SEGMA hospitals. The "free" caesarean section, however, amounts to an average of US$169 (95% CI: 153-185) at SEGMA hospitals and US$291 at the UH. This extra cost may jeopardize the fee exemption policy. Can this cost be reduced? The cost of purchase of pharmaceutical products could be lower. The prescriptions on leaving the hospital show that currently the prescribed drugs are, or can be, available in the hospital pharmacies. The total amount paid for prescriptions on leaving the hospital is inflated by the costs of prescriptions for anticoagulants. These are systematically prescribed in SEGMA hospitals. They are administered during the hospital stay (2 or 4 injections) and prescribed on leaving (6 injections). The issue of whether these are really necessary remains an ongoing debate. At the UH, prescription of anticoagulants is not systematic: only 50% of women were prescribed this treatment. The cost of the prescribed drugs was around US$85 ± 45 in the SEGMA hospitals, nearly half the direct expenses of the households that sometimes are unable to pay for the complete treatment. This is also the case elsewhere, where drug expenditures represent a significant portion of expenses, ranging from 18 to 55% for most countries and even reaching 70% in Bangladesh and India [11]. Sometimes, patients could not take prescribed medication because they could not get it due to stock shortages [12].

In the direct costs of a caesarean section, the total transport cost, which on average amounts to US$52 ± 31 for the SEGMA hospitals, represents nearly one third of the expenses for the family. The big part of this cost, however, comes from the journeys made by the husband who needs to visit his wife every day; this part can hardly be covered by the policy. The transport by ambulance had to be paid for in a third of the cases (4 out of 12) as no ambulance from the Ministry of Health was available, although the ministerial decision included transport with an ambulance from home in the rural areas and between the peripheral maternity and the hospital for women who needed to be referred. In other countries this is equally an important cost and ranges from 2.23 US$ in Benin to 59.94 US$ in Bangladesh for a complicated delivery [13].

In areas of difficult geographical access, it becomes particularly important, averaging between US$15 and 41, as in Nepal where transport accounts for over 50% of the cost of a normal delivery, and 25% of the cost of complicated childbirth [14]. In Cambodia, the cost of transportation is the biggest barrier, even more than the costs of care in health facilities [15]. The cost of transportation accounted for 71% of total costs in Tanzania [16].

The cost of extra food and the opportunity costs were not significant in this study. Internationally, the loss of income of the companions was significantly higher in the case of a complicated delivery: US$4.13 (in Bangladesh) to US$78.5 (in Tanzania). The opportunity cost in Ghana was also 5% of total costs [13].

The cost of tips and informal payments, which averaged US$20 ± 12 in SEGMA hospitals (for the 46% of people who said they made such payments) does not represent a significant expense. The ethical weight of this cost is higher than the financial weight, since it represents only 6% of direct costs of a caesarean section in SEGMA hospitals and 1% in the UH. Flexible visiting hours, the time allowed for these visits, the opportunity for the father or the family to take the baby instead of the hospitalized mother keeping it, the quality of meals served and the professional attitude are all factors that fuel the practice of informal payments to obtain rights and privileges. This calculated average is minimal compared to other countries where informal fees can be substantial. In India, they are 5 times more important than administrative costs and account for 80% of total direct spending by households [17]. In Kenya, they represent 59% [18]. The practice of informal payments is common and applies to all categories of medical, paramedical and administrative staff. It was estimated at about US$20 per hospitalization in Madagascar [19].

The inclusion of the UH in the fee exemption system needs to be resolved urgently. In fact, UH users paid an average administrative cost of US$138(95% CI: 98-178), which almost doubles the cost of a caesarean section. Most women, however, were referred to the UH without necessarily having had a choice.

As a result of the fee exemption policy, the reduction in direct costs for women delivered by caesarean section in SEGMA hospitals amounts currently to US$114 on average (compared with the US$283 paid on average before). This is a reduction of average direct costs of 40%. Extreme amounts paid range from US$26 to US$ 376, this cost taking into account the intervention and the hospital stay which may vary between 4 days and 1 month according to the complications. This 40% average reduction is higher than the one recorded in Ghana, where a 28% decrease was reported in the average cost of caesarean deliveries and after the policy of fee exemption was introduced [20]. In another study, the reduction in direct costs for women amounted to between 195 US$ and 153 US$ for caesarean sections [21].

Our results can also be interpreted more positively when we look at the percentage of the fee exemption in the SEGMA hospitals in comparison to the average direct cost of a caesarean section. The reduction even exceeds 80% of the cost when the patient receives free medication.

This is a major benefit in terms of reduction of family expenditure. However, our study was not able to show whether the fee exemption benefited the poorest families.

## Conclusion

The free caesarean policy is formally implemented in the public hospitals of Fez but that does not make caesareans actually free. Indeed, extra cost for medicines, transport and care in the university hospital in case of referral are not fully covered. The government's effort is certainly commendable but should be reassessed soon to decide whether the fee exemption policy helps the poorest and whether the medicalization it brought about is financially sustainable.

### Endnotes

^a ^e.g. Augmentin^©^, Synthocinon^©^, Intranule^©^, Vicryl^© ^surgical thread, TardyfèronB9^©^, human insulin, Doliprane^©^, syringes, serum, thermometer, Adalate^©^...

## Competing interests

The authors declare that they have no competing interests.

## Authors' contributions

IB contributed to the design of the study, collected all the data, including interviews, analyzed the data and wrote the first draft of the paper. VDB contributed to the design of the study, the interpretation of data analysis and finalized the writing. Both authors read and approved the final manuscript.
